# Significance of Algal Polymer in Designing Amphotericin B Nanoparticles

**DOI:** 10.1155/2014/564573

**Published:** 2014-11-12

**Authors:** Saurabh Bhatia, Vikash Kumar, Kiran Sharma, Kalpana Nagpal, Tanmoy Bera

**Affiliations:** ^1^PDMCOP, Bahadurgarh, Haryana 124507, India; ^2^Department of Pharmaceutical Sciences, Jamia Hamdard, New Delhi 110062, India; ^3^School of Pharmacy, Faculty of Applied Medical Sciences, Lovely Professional University, Phagwara, Punjab 144411, India; ^4^Department of Pharmaceutical Technology, Jadavpur University, Kolkata 700032, India

## Abstract

Development of oral amphotericin B (AmB) loaded nanoparticles (NPs) demands a novel technique which reduces its toxicity and other associated problems. Packing of AmB in between two oppositely charged ions by polyelectrolyte complexation technique proved to be a successful strategy. We have developed a novel carrier system in form of polyelectrolyte complex of AmB by using chitosan (CS) and porphyran (POR) as two oppositely charged polymers with TPP as a crosslinking agent. Initially POR was isolated from *Porphyra vietnamensis* followed by the fact that its alkali induced safe reduction in molecular weight was achieved. Formulation was optimized using three-factor three-level (3^3^) central composite design. High concentration of POR in NPs was confirmed by sulfated polysaccharide (SP) assay. Degradation and dissolution studies suggested the stability of NPs over wide pH range. Hemolytic toxicity data suggested the safety of prepared formulation. *In vivo* and *in vitro* antifungal activity demonstrated the high antifungal potential of optimized formulation when compared with standard drug and marketed formulations. Throughout the study TPP addition did not cause any significant changes. Therefore, these experimental oral NPs may represent an interesting carrier system for the delivery of AmB.

## 1. Introduction

**Figure 1:**
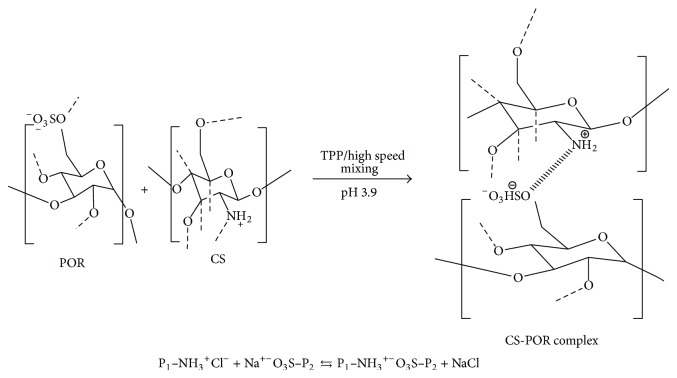
Possible interaction between CS and POR under the influence of the specified conditions.

Increasing prevalence of serious systemic infections such as aspergillosis, candidiasis, and cryptococcosis demands a potent fungicidal agent that effectively destroys the fungal growth without the development of any resistance and toxicity. Amphotericin B (AmB) is a broad-spectrum polyene macrolide antifungal agent that does not induce resistance, is widely known for the treatment of life-threatening systemic fungal infections, and acts as second line drug of choice for visceral leishmaniasis [1–4]. However its poor water solubility, poor stability (in acidic pH), low intestinal permeability, and various dose related serious side effects, for example, nephro and hemolytic toxicity, limit its therapeutic efficacy in oral drug delivery system [5]. All these problems are associated with different states of AmB in aqueous media which determines the overall activity of drug [5]. That is why it is conventionally administrated parenterally. We have effort fully established a clear relationship between the solubility, antifungal activity, and its related toxicities (Figure 1). Nanoparticulate delivery system is the most suitable mode for delivering AmB. Most of the currently available AmB nanoformulations are lipid based (Ambisome, Amphocil, and Abelcet) though some are also available in micellar (Fungizone) and nanosuspension form [2]. These all formulations have their serious concerns such as rapid release, surfactant related toxicities, low drug loading capacity, difficult route of administration, limited* in vivo* efficacy, and high price [2]. Thus there is an urgent need for effective oral antifungal drug delivery system that not only reduces the side effects but also increases the absorption of AmB in a controlled manner.

PEC technique for nanoparticles involves the controlled mixing of diluted polycation and polyanion solutions that gives the size range of 20 to 500 nm with various shapes such as spherical, toroid, and rod-like shapes or have a loose gel-like shape up to compact internal structure. They are easily prepared (usually does not require any stabilizer or surfactant) and economic and are nontoxic in nature. PEC particles can serve as carrier for low (drug) to high molecular weight (protein) compounds. They are efficient in binding or internalization at various types of human cells. One of the major problems associated with PEC is their strong aggregation tendencies. Usually this type of aggregation has sufficient colloidal stability which can be modulated by polyelectrolyte concentration, ionic strength, pH, polyelectrolyte structure, and molecular weight [6, 7].

**Figure 2:**
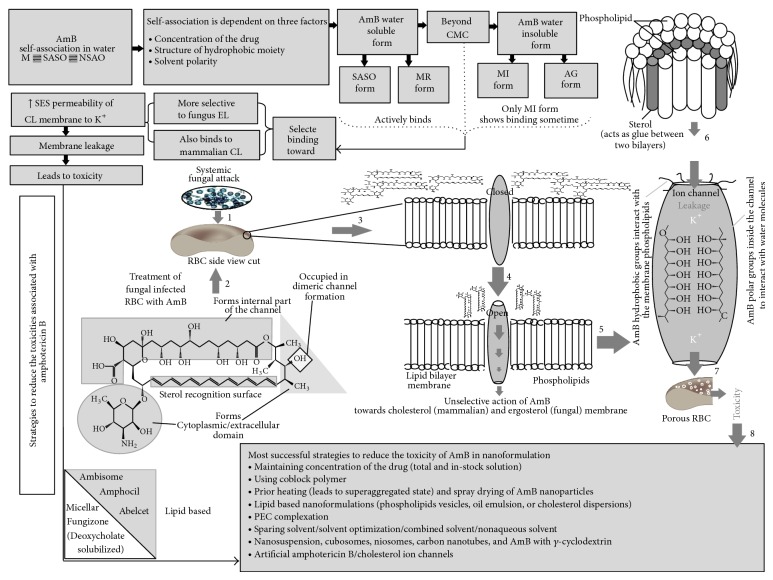
Mechanism of antifungal action of amphotericin B representing its relation with the nature of arrangement (form) in aqueous state with its associated toxicities and cures. This hypothetical figure demonstrates how association of AmB affects antifungal activity of the whole dug. AmB, oldest drug that does not induce resistance, possesses poor solubility in water (soluble in some organic solvent, e.g., DMSO/DMF) but beyond critical micellar concentration (CMC) AmB starts self-association in aqueous media. This type of association in aqueous media creates the equilibrium stage (between monomers (M), self-associated soluble oligomers (SASO), and nonsoluble aggregation of oligomers (NSAO)) which is dependent on several factors as depicted in Figure 2. Beyond CMC, the solubilized form (monomeric (MR) and self-associated oligomer) is converted into insoluble form aggregated {(AG)/miccellar (MI) form}. Therefore due to availability of several forms of single drug it attains different types of activity. The soluble form can be an active form since it actively binds to the membrane either at once or after reconstitution in micelles within the lipid bilayer but it is only possible beyond CMC. Among insoluble, the micellar form can be active in some cases. As a result the overall activity of AmB is dependent on the equilibrium stage between the different forms present in the aqueous medium. Factors influencing this stage can change the whole activity of the drug. Among these two forms, soluble form (self-associated oligomer) effectively/unselectively binds with the fungal ergosterol membrane and cholesterol membrane by increasing permeability to K^+^ but proved to be more toxic than aggregated form as it causes leakage to mammalian cholesterol also. This leakage is governed by formation of AmB-sterol complex in a fashion where polar groups head towards the inside of the channel and hydrophobic groups interact with the outside phospholipid membrane. This may lead to various toxicity problems. Nowadays various strategies have been adopted to reduce these toxicities while formulating AmB in nanoform [8–14].

Biodegradable polymeric nanocarriers have attracted a lot of attention towards drug delivery for hydrophobic drugs. They are reported as the best candidates for delivering optimum drug with increased absorption at targeted site. Loading of these drugs in form of polyelectrolyte complex was proved to be a good strategy to control the drug release rate and to improve their bioavailability [7]. Therefore due to established advantages and suitable features of two biopolymers, CS and POR are selected for present study for the formulation and development of AmB. The possible interaction between the two oppositely charged polyions is presented in Figure 2.

Due to various appealing properties such as biodegradability, biocompatibility, low toxicity and relatively low production cost, and hydrophilic nature, CS is widely used as a polymeric drug carrier material in several dosage forms. Fast dissolution at low pH and insolubility above pH 6 hinders some of its applications in pharmaceutical field. Chemical modification such as copolymerisation or derivatisation helps in improving its properties but they may also lead to the formation of new chemical entities with unknown toxicological profiles. Thus, physical modification of the polymer is preferred compared to chemical modifications. Formation of stoichiometric polyelectrolyte complexes by addition of polyanion is an excellent strategy to overcome these problems. Currently most of the researchers are focusing towards discovery/selection/exploration of natural polyanion which could not only be safe, biodegradable, biocompatible but also form stable complex without or with least amount of crosslinking agent [6, 18].

*Porphyra*, red algae, contains high amount of active components (e.g., mycosporine amino acids, POR, pigments, fatty acids, etc.). There are several factors (type of the species, season of collection, and isolation procedure) that influence the yield of active components of* Porphyra* [19, 20]. POR is a polyanionic SP which is biodegradable, biocompatible, and linear branched negatively charged polymer containing a chain of anhydroglucose units and contains approximately 17–30% sulfur [20–22]. Due to efficient biological and pharmaceutical properties, POR has recently shown extensive applications in drug delivery [23]. So far various SPs such as chondroitin and dextran sulfate, fucoidan, and carrageenan have been introduced with chitosan as PEC in nanoparticulate form [24–28]. These evidences created more interest in formulating new SPs as more stable and effective PEC. Hence in this work POR was introduced as a polyanion which can work in a dual fashion firstly by providing polyanionic group to CS (polycationic) to form stable stoichiometric complex of nanoparticulate system which delivers the optimum concentration of AmB at desired site and secondly by synergizing the antifungal activity of drug due to presence of sulfate group.

Various formulation and process variables, namely, concentrations and ratio of polymer/drug/surfactant, mixing time, speed, pH, homogenization speed and time, and so forth, influence the characteristics of nanoparticulate delivery systems. The concept of mathematical modeling and statistical approach of optimization has been considered more powerful than traditional methods of changing one factor at a time for multifactor optimization. Central composite, an independent quadratic design which does not contain any embedded factorial or fractional factorial design, is applied here to optimize the process. The electrostatic interaction between both polymers was checked by DSC thermogram and FTIR spectra [29–31]. Thus our objective was to prepare and characterize AmB loaded CH-POR polyelectrolyte complex nanoparticles and evaluate its* in vivo* and* in vitro* antifungal activity against CH-AmB nanoparticle.

## 2. Experimental

### 2.1. Test Organisms

Pathogens, namely,* Candida albicans* (ITCC 4720),* Aspergillus fumigates* (ITCC 4448),* Aspergillus flavus* (ITCC 6353), and* Aspergillus niger* (ITCC 5448), were procured from Indian Type Culture Collection, IARI, Delhi. These pathogens were cultured in the laboratory using Sabouraud dextrose (SD) agar plates and inoculated with stock cultures of* Candida* and* Aspergillus* strains followed by the BOD incubation at 37°C. 72 h cultured strains were harvested and homogeneously suspended in phosphate buffer saline (PBS). Before performing the experiments spores were counted by using haemocytometer and the number was adjusted to 1 × 10^8^ spore/mL for* Aspergillus* strains and 1 × 10^3^ spore/mL for* C. albicans*.

### 2.2. Raw Material

*P. vietnamensis* was collected during high tide from Maharashtra coast. Natural polysaccharide (porphyran) was isolated and purified by modified Ishihara et al. (2005) method [31]. The obtained yield was 28.9%; however yield is strictly dependent on the season of collection and isolation procedure. Total ash content (determined by oxidizing* Porphyra* at 450°C), protein content (by Lowry method) [32], total sugar content (by phenol sulfate method [33]), galactose and 3, 6-anhydrogalactose (AGR) contents (by resorcinol method) [34, 35], and sulfur content (by toluidine method) [36] were found to be 1.4%, 11.3%, 71.4%, 47.2%, 21.1%, and 16.36%, respectively. The sulfate and AGR are the main components of this polysaccharide. The purified sample is treated with lowest concentration of sodium borohydride (NaBr). Two samples (natural porphyran (NPOR) and NaBr treated (POR)) were analyzed (FTIR and DSC) to check how physicochemical properties of POR affect the formulation and development of AmB.

### 2.3. Materials

AmB oral grade (Batch no. 13A01N01) was supplied by Synbiotics (India) as a gift sample, AmB parenteral grade was purchased from Sigma Aldrich, India. Native porphyran (NPOR MW 780,000 da), Alkali modified porphyran (POR MW 33,000 da), Chitosan was procured (Central Institute of Fisheries Technology, Cochin, No. F.1 (2)/BP/Chitin/12) and the molecular weight CS 39,000 da was reduced, with 95% deacetylation); NaBr and Tween 80 were purchased from Sigma Aldrich (USA). Dialysis membrane bag (Himedia, MWCO, and molecular mass cut-off 12000–14000, pore size 2.4 nm). Simulated gastric fluid (SGF) was prepared as per USP XXIX. Double distilled water was used in all the preparations. All other solvents and chemicals used were of analytical grade.

### 2.4. Preparation of CS-POR PEC Nanoparticles

Various methods for the preparation of AmB loaded NPs have been developed. In present study AmB NPs were prepared by polyelectrolyte complexation method [25]. AmB (20 *μ*L) in dimethylsulfoxide (DMSO; 10 mg/mL) was added to 0.075 mL of aqueous solution of POR and mixed with 0.755 mL of deionized water. The solution obtained was stirred continuously at 600 rpm. Aqueous CS solution was added dropwise (0.1 mL) to the resulting solution. Then 50 *μ*L of tripolyphosphate (TPP) solution was added. The resulting stabilized AmB NPs were dialyzed. Similar procedure was again repeated for CS-POR NPs (without TPP) and CS NPs (without POR and TPP). The nanosuspension obtained was freeze-dried (prefreezing at −20°C in deep freezer) (Martin Christ model Alpha 1-2 LD* plus*) at −55°C, 0.01 mm of Hg using Mannitol (5% w/v) as a cryoprotectant. Limits and ranges for CS, POR, and TPP were set for the optimization studies using a central composite design (CCD) by three factor (3) at three-level: *X*1 (i.e., percent CS concentration; w/v), *X*2 (i.e., percent POR concentration; w/v), and *X*3 (i.e., percent TPP concentration; w/v). These conditions were adopted for further investigations as required by the design, and the factor levels were suitably coded.  Table 1 summarizes the 24 experimental runs studied employing different 10 levels of the three factors.

### 2.5. Optimization Data Analysis

Design Expert Software, Design Expert 9.0 (Stat-Ease, Minneapolis, MN, USA), was employed to fit full second order polynomial equations with added interaction terms to correlate the studied responses with the examined variables. The response variables for systematic optimization were particles size (nm), zeta potential, and PDE (maximum percent drug entrapment). The optimum formulations prognosis was conducted by locating feasible space and secondly, an exhaustive grid search was conducted to obtain the possible solutions. The optimum solution was also located by the software using the overlay plot. The optimized drug loaded CS-POR nanoparticles batch was utilized for further* in vivo* and* in vitro* studies [30].

### 2.6. Particle Size, Zeta Potential, and PDI

Size distribution and zeta potential of drug loaded CS-POR PEC NPs were evaluated by using Zetasizer [Nano-ZS90 (Malvern Instruments Limited, UK)]. All measurements were carried out after 10 times dilution of NPs with HPLC grade water.

### 2.7. Percentage Yield of Nanoparticles

The % yield of NPs was determined by washing unentrapped drug from drug loaded prepared NPs with water (thrice). Washed NPs were redispersed in water and subjected to lyophilization. Lyophilized NPs were analyzed for yield. The NPs yield was calculated by using the following equation:

(1) %  yield of nanoparticles    =Mass of nanoparticles recoveredMass of all ingredients×100.

### 2.8. Percent Drug Entrapment (PDE) Efficiency

AmB loaded NPs were centrifuged at 12,000 ×g for 20 min. The supernatant was decanted and saved for CS and POR concentration determinations. The pellet was dissolved in 0.5 mL of DMSO and then centrifuged at 12,000 ×g for 20 min. 20 *μ*L of the supernatant was mixed with 0.98 mL of methanol : water (1 : 1) solution. The amount of AmB was determined from its absorption at 408 nm. The sample concentration was then obtained from a calibration curve from 1.6 to 4.8 mg/mL of AmB in 50% v/v methanol [37].

The percentage of drug entrapped was then calculated from

(2) %  drug  entrapment  =Amount  of  drug  present  in  nanoarticlesTotal  amount  of  drug  added×100.

### 2.9. Drug Loading Parameters

For drug loaded amount (LA) determination AmB loaded NPs were destroyed via the addition of 0.15 mL/mL trichloroacetic acid (TCA, 2 N) and subjected to vortex mixing (5 min) and centrifugation (12,000 ×g for 5 min). After that 20 *μ*L of supernatant was then injected into the HPLC system. Finally, the loading parameters of the drug in NPs were calculated as

(3) %  LE =AmB  loaded  amount  LAtotal  AmB  added  during  the  loading  procedure  ×100,%  LC =AmB  loaded  amount  LApolymer weight+AmB loaded amount×100.

Here in this study the sum of polymer weight and AmB loaded amount was approximately considered as the total NPs weight.

### 2.10. UV-VIS Spectroscopy Characterization of AmB in Nanoparticles

It has been reported that aggregation of AmB plays a vital role in studying their toxicity profile. The overall activity of AmB is dependent on its actual state (monomeric, dimeric, self-associated oligomeric, aggregated, polyaggregated, superaggregated, and micellar form) present in nanoformulation. Toxicity profile of AmB varies with its different forms. Hence it is worthy to determine the actual state of AmB in test samples. UV-VIS spectrophotometric method was used to determine the actual state of AmB in the test samples. Two marketed preparations (Fungizone and Ambisome) along with plain AmB in 50% methanol were taken as reference compounds. For UV characterization AmB NPs in lyophilized form were weighed initially and then dispersed in 5.0 mL of DI water to give final AmB concentration of −10 *μ*g/mL. Using an absorbance range from 300 to 450 nm, the UV-VIS spectra were obtained. Unloaded NPs were used as a blank [25].

### 2.11. Transmission Electron Microscopy (TEM) Characterization

Optimized formulation was characterized for its morphology using transmission electron microscope (Morgagni 268 D, Fei Co., The Netherlands, operated at 60 KV). The samples (Karnovsky's fixed) were washed in phosphate buffer (0.1 M, pH 7.4, 6°C) and postfixed for 2 h in 1% osmium tetroxide in the same buffer at 4°C. Samples were sonicated in order to prevent aggregation. Sonicated sample was fixed and then dehydrated. The NPs samples were stained with uranyl acetate and lead acetate and then examined under transmission electron microscope.

### 2.12. *In Vitro* Dissolution Studies

Due to very poor aqueous solubility of AmB 1% (v/v) Tween 80 in 10 mM phosphate-buffered saline (PBS, pH 7.4) buffer, pH 7.4, was used as a dissolution medium and* in vitro* drug release of AmB studies was performed at 37 ± 0.5°C using the equilibrium dialysis technique. Under dark and continuous shaking, NPs (equivalent to 1 mg AmB) were placed into dissolution medium (5 mL) and then placed in a dialysis membrane bag. The membrane bag was placed in 300 mL PBS (37 ± 1°C). The 50 rpm was set to avoid the excessive torque produced at higher rpm which may otherwise rupture the dialysis membrane. At predetermined time intervals, 5 mL aliquots were collected and equal volume of fresh PBS was replaced into release system. After filtration of collected samples through a 0.2 *μ*m membrane, the sample was evaluated for the concentration of AmB using UV Visible Spectrophotometer.

### 2.13. Differential Scanning Calorimetry (DSC)

The thermogram of N-POR, POR, CS, AmB, TPP, mannitol, optimized batch of AmB loaded CS-POR nanoparticles, and CS-POR blank NPs was obtained using DSC (Model Q10, TA Instruments Pvt. Ltd., USA) and then an overlay was prepared using the in-built software. The samples were sealed in the aluminum crimp cell and heated at the speed of 10°C/min from 40 to 400°C in nitrogen atmosphere.

### 2.14. Sulfated Polysaccharide Assays

The supernatant (POR and CS) collected during the evaluation of drug entrapment efficiency was utilized for sulfated polysaccharide determination. For the complete dissolution of polyelectrolyte complex the isolated supernatant was mixed with 1 M hydrochloric acid at 25.0 *μ*g/mL and dissolution was confirmed by DLS analysis. Completely dissolved samples were mixed with dimethylmethylene blue (DMB) and evaluated by spectrophotometry at 525 nm. The sulfated polysaccharide concentration in the NPs was calculated using a calibration curve in triplicate [28].

### 2.15. Stability of AmB in Simulated Gastric Fluid

Oral and parenteral plain AmB (10 mg) were dissolved in simulated gastric fluid (pH 1.2) and incubated in 50 mL volumetric flask at 37°C for 6 h. Then CS-AmB, CS-POR-AmB, and CS-POR-AmB-TPP NPs were studied under similar condition for 12 h. The percentage degradation of AmB in SGF was determined at different time intervals, spectrophotometrically, at 272.6 nm [38]. Spectrophotometrically mediated quantification is a direct and simple method; however, selectivity, specificity, precision, accuracy, and robustness are quite lower than HPLC (high-performance liquid chromatography) method. In addition LOD (limit of detection), LOQ (limit of quantification), and range will be good in HPLC compared to spectrometry. In addition many degradation products can maintain absorbance characteristics while they have important chemical structural differences which serve as an important limitation of the simple spectrophotometry. Therefore simple spectrophotometry is not enough to guarantee nondegradation. However, UV spectrophotometry method is quite easy and simple and does not need the elaborate treatment and procedures usually associated with chromatographic methods like HPLC. Thus in the present study we opted UV method for dissolution studies.

### 2.16. *In Vitro* Antifungal Test

#### 2.16.1. Preparation of Drug and Nanoparticles Stock Solution

Stock solution AmB was prepared in DMSO (below 0.6% v/v) and Millipore water. AmB NPs were suspended in sterile water in aseptic condition. The stock solutions of marketed preparations (Ambisome and Fungizone) were prepared according to the instruction mentioned on their container/package insert. Two serial dilutions with Millipore water were performed (for both prepared and marketed preparations) in sterile 96-well microtiter plates at 4°C. For disc diffusion assay the concentration of AmB (reference compound) and nanoformulations was tested over the range 0.05–50 *μ*g/mL (5.15 *μ*g/mL to 50 *μ*g/mL for the hemolysis assay).

#### 2.16.2. Disc Diffusion Assay

The antifungal potential of the prepared AmB NPs was evaluated by using disc diffusion assay (using radiation sterilized petriplates, 10.0 cm diameter, Tarsons). The assay was performed by exposing fungal strains of* Candida albicans* and* Aspergillus* spp. to the increasing concentrations of NPs which determines MIC value that prevents detectable growth in test wells. The prepared SD agar plates were plated with standardized suspension of* Aspergillus* spp. (1 × 10^8^ spore/mL) and* C. albicans* (1 × 10^3^ spore/mL). CS-POR blank NPs were treated as control. The inoculated plates were dried and discs ((5.0 mm in diameter) of Whatman filter paper no. 1) were placed on the surface of the agar. The test sample/reference/marketed stock solution preparation procedure and its tested concentrations are mentioned above. After that reference compound and NPs preparation were diluted and added to the wells. The plates were incubated at 37°C and examined at 24 h, 48 h for determining zone of inhibition around the discs. The concentration which developed the zone of inhibition of at least 6.0 mm diameter was taken as end point (Minimum Inhibitory Concentration; MIC). Otherwise no growth inhibition was declared. All experiments were performed in triplicate [39].

### 2.17. Hemolytic Assay

Healthy human erythrocytes were isolated from fresh whole blood and erythrocytes were removed by centrifugation at 5000 rpm for 5 min at 4° ± 1°C. The centrifuged erythrocytes were resuspended in normal saline solution to a 10% hematocrit value. After removing the supernatant the RBCs pellet was lysed with sterile water (considered as 100% hemolysis) and normal saline was taken as blank for spectrophotometric estimation at 540 nm. After that RBC suspension was treated with different dilutions of AmB loaded preparations (dilutions were made in 4.5 mL of sterile water) including standard drug (AmB), marketed preparations (Ambisome and Fungizone), and prepared formulations (CS-POR, CS-AmB, CS-AmB-POR, and CS-AmB-POR-TPP). Same procedure was repeated again by treating 0.5 mL of drug solution and 0.5 mL of blank CS-POR formulation. Both were mixed in 4.5 mL of normal saline and allowed to interact with the RBCs suspension. The CS-POR and AmB were taken in separate tubes at equivalent concentrations. The concentration of AmB (reference compound) and nanoformulations was tested over the of range 5.15 *μ*g/mL to 50 *μ*g/mL for the hemolysis assay [40].

Percentage hemolysis was determined for each sample as follows:

(4) %  Hemolysis=Absorbance of the sampleAbsorbance of the control  without formulation×100.

### 2.18. *In Vivo* Toxicity

All preparations in form of single dose injections (1, 4, and 8 mg/kg AmB) were given to 5–8 male mice weighting 30–40 g. Empty CS and CS-POR NPs at equivalent amount of CS and POR in AmB-NPs (CS-POR-TPP was only administered* in vivo* hematological test) were also injected into the mice as negative control. After the collection of blood at 14th day from survivor mice the following parameters were evaluated in two separate studies. To determine the* in vivo* hematological toxicity of the prepared formulations various parameters such as hemoglobin concentration, hematocrit percentage, white blood cell (WBC), red blood cells (RBCs), and platelets count were evaluated by using standard methods [41]. In order to determine the nephrotoxicity and kidney function, blood urea nitrogen (BUN) and serum creatinine were studied. The obtained results of each group of treated mice were compared with those of the control groups using one-way analysis of variance (ANOVA). Significant level was defined as *P* < 0.05 [25].

### 2.19. Statistical Analysis

All study data are reported as mean ± SEM (*n* = 6). The difference between the groups was tested using analysis of variance (ANOVA) followed by Tukey's post hoc test for biochemical parameters at the level of *P* < 0.05 using GraphPad Instat 3 (GraphPad Software, Inc. San Diego, CA). The difference greater than *P* < 0.05 was considered significant.

## 3. Result and Discussion

### 3.1. Preparation and Optimization of CS-POR PEC NPs

The NP preparation method was based on the ionization of CS and POR polysaccharides carboxyl and sulfate groups, which interact efficiently with the positively charged CS under controlled conditions (as demonstrated in Figure 2). Due to the higher viscosity of CS based POR solution, a number of experiments were performed by varying the concentration of CS and POR. This was done to screen the appropriate concentration range, pH, and mixing ratio of polymeric solutions yielding turbid solution without aggregation. A direct relationship between the particle size and the CS concentration was observed in preoptimization studies. Variation in POR concentration also affects the particle size and PDE. Moreover the concentration of TPP influenced the entrapment efficiency and dissolution of drug though the results was not significant when compared against CS-POR NPs. The final concentration range selected for optimization study using central composite design was 0.01–0.07% w/v of CS, 0.02-0.03% w/v of POR, and 0.01–0.04% w/v of TPP. At a concentration of 0.01 and 0.02% w/v of CS, the particle size of the NPs was found to vary between 111 nm and 133 nm. At 0.04% concentration of CS the size of NPs varied between 158 and 255 nm. Similarly for 0.06-0.07% the size varies between 240 and 360 nm as tabulated in Table 1.

**Figure 3:**
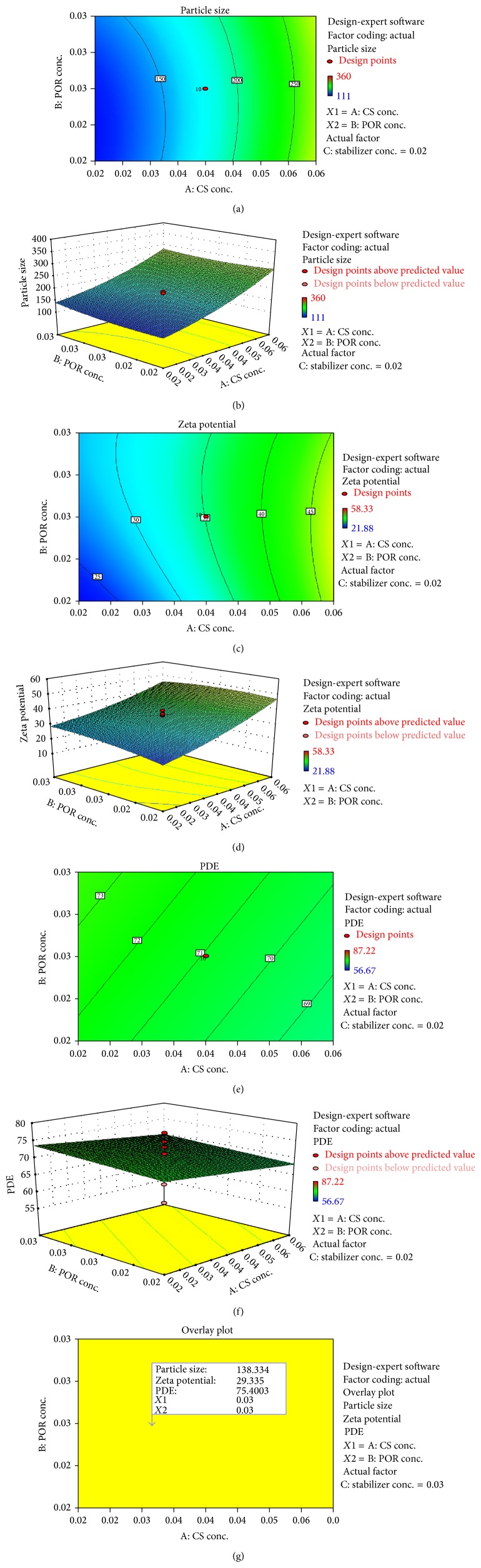
Response surface graph of CS-POR nanoparticles encapsulating AmB showing the effect of chitosan and porphyran on (a) two-dimensional particle size, (b) three-dimensional particle size, (c) two-dimensional zeta potential, (d) three-dimensional zeta potential, (e) two-dimensional DEE, (f) three-dimensional DEE, and (g) overlay plot showing the location of optimized formulation.

Exploration of effect of independent variables on characteristics of NP using response surface methodology. Only *X*1 and *X*2 significantly affected the particle size of nanoparticles in the range studied in the present investigation. By increasing the concentration of CS from 0.02% to 0.06%, the particle size increased while POR from 0.02 to 0.03% decreased the particle size (Figures 3(a) and 3(b)), whereas chitosan from 0.02% to 0.06% elevated the zeta potential up to certain level (Figures 3(c) and 3(d)). The following equation was generated for quadratic model using Design Expert software:

(5) Particle  Size  =176.4295+75.4029X1+6.044225X2   −27.6882X3−5.125X1X2−10.375X1X3   −9.375X2X3+19.55021X12+8.766834X22   +9.297165X32Quadratic  equation.

CS (at conc. 0.02% to 0.06%) and POR (at conc. 0.02 to 0.03%) increased the zeta potential:

(6) Zeta potential  =35.11391+10.22095X1+1.207641X2   −3.68792X3−1.89125X1X2−1.55625X1X3   −1.29875X2X3+2.269644X12−1.65657X22   −0.30069X33Quadratic equation.

Figures 3(e) and 3(f) show the effect of CS concentration and POR concentration on PDE as contour plot and response surface, respectively. The increase in concentration of CS decreased PDE whereas on increasing the concentration of POR PDE was increased. The decrease in PDE may be attributed to higher leaching of the drug through CS network as the POR may increase the drug permeability:

(7) PDE=70.9275−1.73905X1+0.935286X2 +5.36401X3  Linear  equation.

Overlay plot (Figure 3(g)) was utilized for feasibility range search with a goal to minimize response 1, that is, particle size (ranging between 111 nm and 360 nm; maximum importance was given to particle size), and to maximize response 2, that is, PDE (which ranged between 60 and 87%; least importance was given to PDE), and suggested a particle size (138.334 nm), zeta potential (29.33 ± 2.85 mv), and PDE (75.40%) using 0.03% w/w CS (Factor *X*1) and 0.03% w/w POR (Factor *X*2). The optimized formulation (0.03% w/w CS and 0.03% w/w POR) was also formulated and evaluated. The average values of particle size, zeta potential, and PDE were found to be 135.18 ± 7.3 nm, 27.11 ± 1.64 mv, and 78.71 ± 6.8%, respectively. Furthermore, the estimated values for loading capacity (%w/w), loading efficiency (%w/w), and polydispersity were 0.37 ± 0.018, 38.12 ± 1.1 (%w/w), and 51.62 ± 2.3 (%w/w). It has been reported that increase in initial drug concentration may increase the drug loading and drug loading should be high enough for any nanoparticulate system to reduce the total amount of delivery system to be used as the dosage unit. The actual ratio is closer to ratios analyzed in our study (data not shown). Furthermore the average percentage yield of NPs (%w/w) obtained was 64.22 ± 1.80 (%w/w). The percent error of mean between predicted values and observed experimental result was found to be 3.27% (for particle size), 3.22% (for zeta potential), and 4.22% (for % PDE), indicating the success of formulation optimization model.

### 3.2. Sulfated Polysaccharide Assays

**Figure 4:**
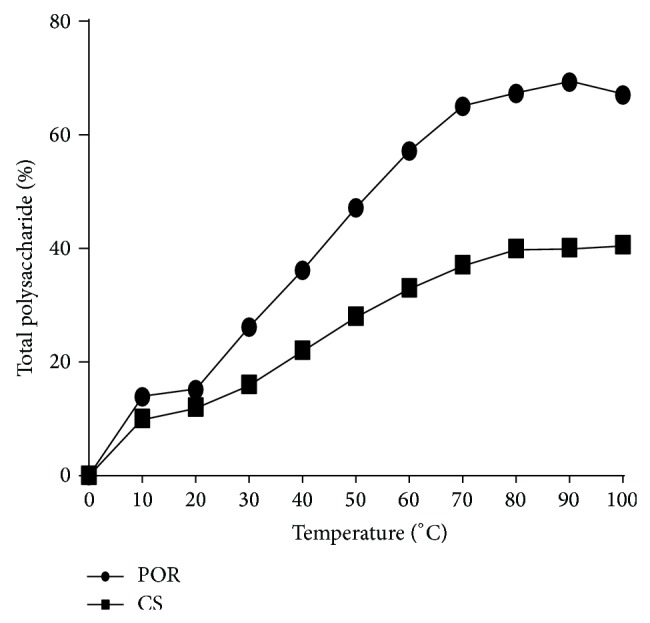
SPs assay of optimized NPs representing the concentration CS and POR at different temperature and acidic conditions by DMB method.

For sulfated polysaccharide determination, the soluble POR and CS were quantified by DMB—metachromatic assay. The POR content in resultant optimized samples was 69.22 ± 0.811 *μ*g/mL (*n* = 13), and CS content was 40.78 ± 0.224 *μ*g/mL (*n* = 3). In this study different prepared formulations were treated at different temperature to study their effect on the content of sulfated polysaccharides in the presence of increasing concentration of acidic hydrolyzing agent (0.2 M, 0.4 M, 0.6 M, 0.8 M, and 1.0 M). Up to 85°C there was a steep increase in POR concentration however no variation in POR concentration has been found after 85°C (1MHCL) (Figure 4). It has been observed that the prepared formulation has shown maximum concentration up to 85°C (at maximum concentration of hydrolyzing agent). But unfortunately after 1M HCL there was slight decrease in concentration followed by constant slop that was observed. Similarly with chitosan a steep increase in concentration was observed up to 80°C followed by constant slope (Figure 4).

### 3.3. UV-Vis Spectroscopy Characterization of AmB in Nanoparticles

**Figure 5:**
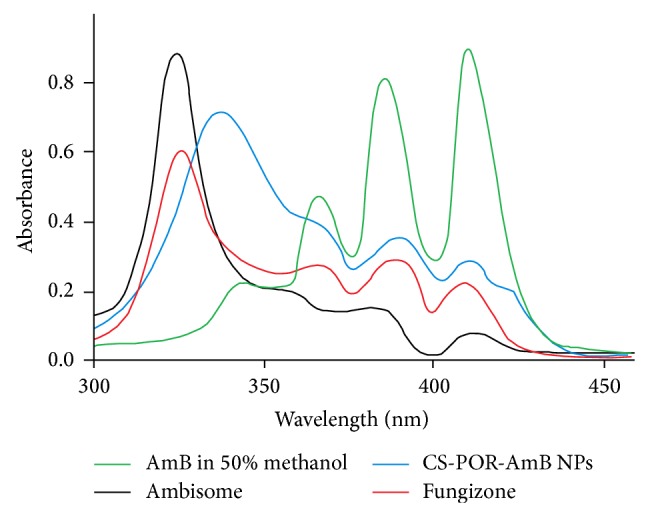
UV/VIS absorption spectra of monomeric AmB (50% methanol), Fungizone, Ambisome, and the developed AmB nanoformulations.

Thus electronic absorption spectrum of AmB is dependent on its aggregation state. The absorption spectrum of aqueous solution of Fungizone represented a major broad peak at 331 nm and less intense peaks at 363, 386, and 412 nm which demonstrated the aggregation of AmB. CS-POR-AmB NPs have shown similar peaks like Fungizone, which indicated the presence of AmB in aggregated state. Similarly, plain AmB in organic solvents (50% (v/v) methanol) showed four peaks with maxima at 359, 371, 388, and 412 nm representing the monomeric form (Figure 5). As far as the toxicity is concerned the aggregated form is usually considered as more toxic than the monomeric form. Determination of peak ratio {absorbance of first peak (I)/absorbance of fourth peak (IV)} assists in representing the spectral changes due to self-aggregation of AmB. Thus in present study this ratio was ranked as follows: Ambisome (4.2) > CS-POR-AmB (3.5) > Fungizone (3.3) > Plain AmB in 50% (v/v) methanol (0.39).

### 3.4. TEM

**Figure 6:**
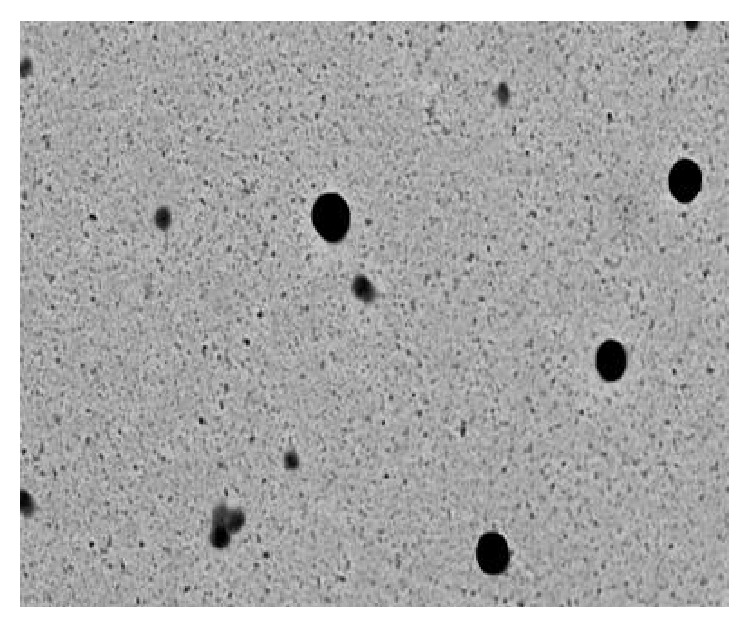
TEM image (18000x magnification) of CS-POR-AmB NPs.

TEM characterization of the optimized batch was shown in Figure 6, revealing that the particle size was uniform and spherical in shape. The particle size of the formulated NPs was less than 200 nm; however a slight variation in size distribution was observed. This might be due to the involvement of uncontrolled charge neutralization process between oppositely charged polyions during the formation of NPs at specific pH. It has been also found that mean particle size of observed NPs was lower than that obtained after Zetasizer. This happened due to the chances of NPs dehydration during sample preparation for TEM analysis.

### 3.5. FTIR Characterization

**Figure 7:**
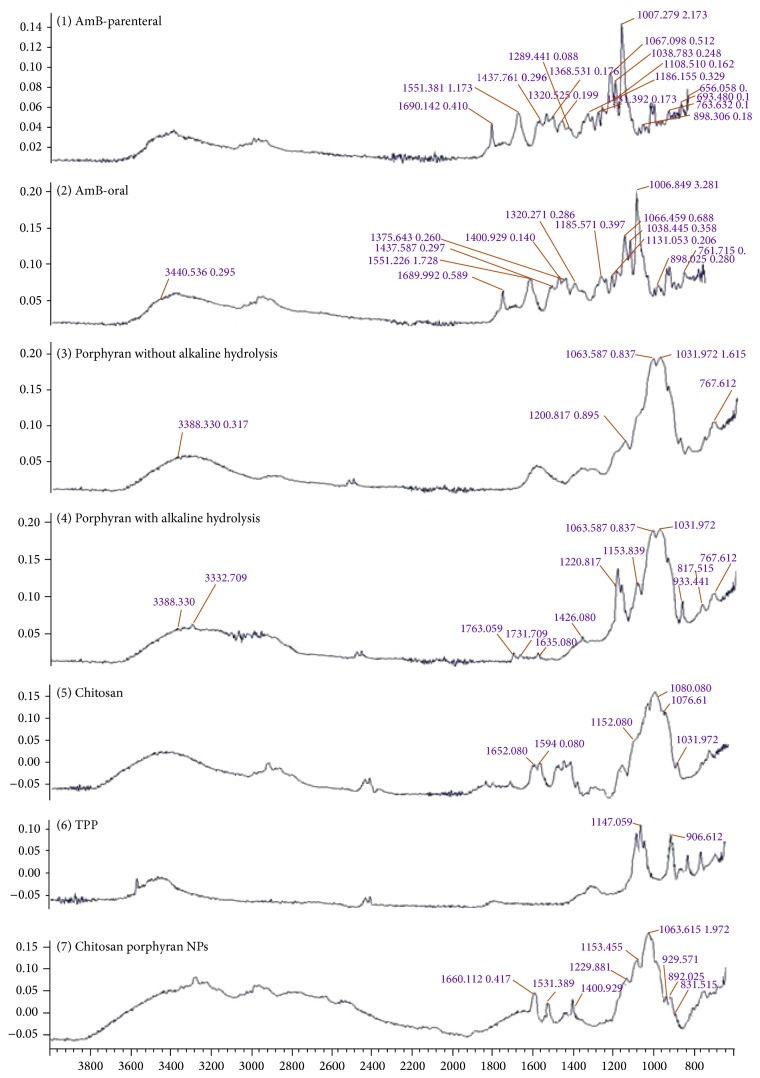
Overlay of FTIR of AmB (oral and parenteral grade), Porphyran (natural and with treatment of NaBr), Chitosan, TPP, and Chitosan-Porphyran-AmB-TPP nanoparticles.

To ensure the similar structural profile of two grades of AmB (oral and parenteral grades obtained from different sources) the FTIR spectra of both samples were obtained. From FTIR analysis it has been proven that both samples are similar and their respective absorption bands belong to the standard absorption spectrum of AmB (Figure 7).

The FT-IR spectra of natural and hydrolyzed POR were shown in Figure 7. For natural sample (NPOR), that is, without treatment of NaBr, the absorption peaks obtained were 3388, 1200, 1063, 1031, and 767 cm^−1^ and the absorption bands after hydrolysis were 3388, 3332, 1763, 1731, 1636, 1426, 1220, 1153, 1063, 1031, 933, 817, and 767 cm^−1^ (Figure 7). Absorption at 1220 and 817 cm^−1^ corresponds to S=O bond stretching and attachment of sulfate group to primary hydroxyl group. The signal at 933 cm^−1^ related with the AGR in the polysaccharide. The presence of both sulfur and AGR unites proves the sample is of POR. Formation of new absorption peaks at 1763 and 1731 cm^−1^ was due to the presence of carboxylic group (Figure 7). Alkali hydrolyzed samples contain equal amount of sulphur group as that of NPOR. Therefore, this procedure could not result in desulfation and hence preserves the biological and pharmaceutical properties of POR whereas on the contrary the AGR was reduced in comparison to NPOR. Thus it has been concluded that the reduced level of AGR could be due to nonselective NaBr hydrolysis of POR which is dependent on the concentration of NaBr used in hydrolysis. It has been also seen that alkaline hydrolysis does not cause major transformation of functional groups in POR sample. This finding justifies that this hydrolysis procedure is safe and maintains structural features of POR though increase in concentration of NaBr leads to the reduction in level of AGR; hence the selected concentration should be minimum [27, 42].

In chitosan absorption spectrum bands at 1652, 1594, and 1080 cm^−1^ correspond to amide I, amide II, and glycosidic linkage (Figure 7). Furthermore absorption bands characteristic of the signals was 1155 cm^−1^ for C–O–C, 1076 cm^−1^ for C–O, and 1032 cm^−1^ for C–O [27].

For TPP the regions from 900 and 1250 cm^−1^ (P=O and P=OH stretching) were selected to confirm the presence of the TPP. Several peaks found in this region could be related to TPP. Furthermore TPP spectrum presents two intense absorption bands at 1147 and 906 cm^−1^ which could be attributed to P=O and P–O along with P–O–P (Figure 7).

It has been observed that the two bands of sulfate and AGR did not appear in NPs spectrum. This might be due to the overlapping of TPP intense peaks with sulfate and AGR of POR. Existence of this overlapping could be accounted on the basis of shifting of the 1147 cm^−1^. Furthermore, the other intense band of TPP disappeared due to shift in 906 cm^−1^ which could be seen in NPs at 892 cm^−1^. The presence of absorption band at 1660 cm^−1^ in NPs corresponds to the adsorbed water bending which could probably masked amide bands of chitosan. The appearance of two strong peaks at 1576 and 1411 cm^−1^ suggested their relation with NH^4+^ bending due to the formation of an ammonium phosphate salt (Figure 7). This finding suggested the interactions between the CS amine groups and the TPP phosphate groups had occurred, which may lead to the formation of NPs [27, 42].

### 3.6. Differential Scanning Calorimetry (DSC)

**Figure 8:**
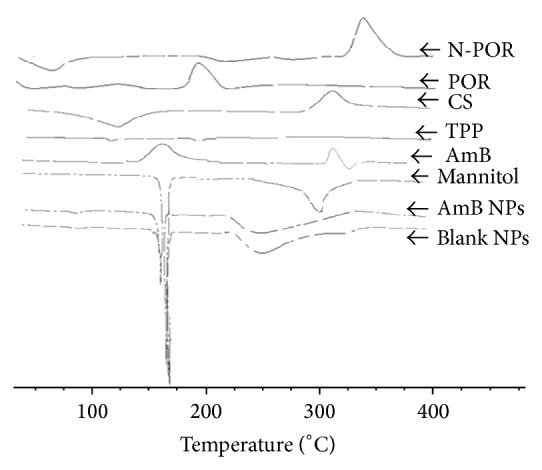
Overlay of differential scanning thermogram of CS (chitosan), porphyran (N-POR, POR), AmB, TPP, mannitol, AmB loaded nanoparticles (AmB NPs), and blank nanoparticles (blank NPs).

DSC thermograms of NPOR (with treatment of NaBr), POR, CS, mannitol, TPP, and optimized AmB loaded freeze-dried CS-POR NP were obtained and an overlay was prepared using the in-built software and shown in Figure 8. Sample NPOR (353.82°C) has shown a sudden shift in the melting point after treatment with NaBr, POR (206.91°C). The DSC thermogram for AmB exhibited two broad endotherms. The drug started to melt >170°C and decomposed (peak at 176.22°C), which is not visible in AmB loaded NP (peaks at 173.33°C and 251.18°C). The blank NP exhibited the peak at 167.77°C and 252.11°C and the cryoprotectant mannitol at 167.29°C.

### 3.7. *In Vitro* Dissolution Studies

**Figure 9:**
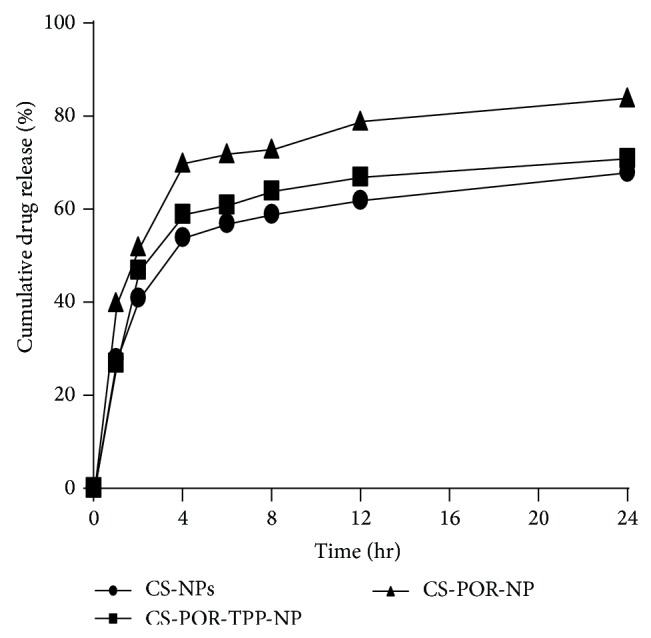
*In vitro* release profiles of the optimized formulations of PEC nanoparticles (CS-NPs, CS-POR-NPs, and CS-POR-TPP-NPs) in PBS buffer, pH 7.4, at 37°C.

Cumulative percentage release of AmB from the optimized batch as per the experimental design was examined at varying time intervals in the dissolution medium, consisting of 1% (v/v) Tween 80 in PBS buffer (pH 7.4) at 37°C. These studies were performed with and without crosslinking agent (TPP). Dissolution studies were also performed simultaneously for AmB loaded CS NPs without POR. The result obtained was shown in Figure 9. The kinetics of drug release was evaluated using various mathematical models for drug release and Baker-Lonsdale Model was the best fit model in both cases indicating the drug release from spherical matrix, further confirming the results obtained from TEM.

### 3.8. Stability of AmB in Simulated Gastric Fluid (pH 1.2)

**Figure 10:**
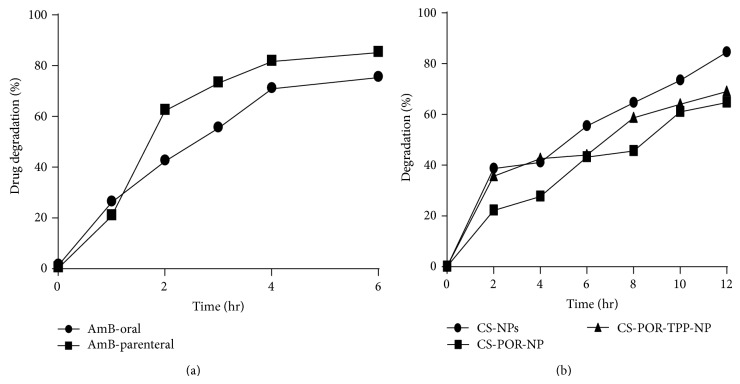
*In vitro* degradation studies of (a) AmB (oral and parental) and (b) optimized formulations (CS-NPs, CS-POR-NPs, and CS-POR-TPP-NPs) in SGF at pH 1.2, (*n* = 3).

For promoting AmB absorption in the GI tract the chemical stability studies for AmB in simulated gastric fluid (pH 1.2) were conducted. These chemical studies were performed at the pH of stomach or intestine to determine the aggregated state (monomeric versus self-associated) of AmB. Stability studies of AmB oral and AmB parenteral in SGF (pH 1.2) showed that the AmB parenteral degraded up to 85% (*n* = 2) whereas oral AmB have shown degradation up to 70% (*n* = 3) in 4 hrs (Figure 10(a)). In contrast with the percentage of degradation of plain AmB and CS NPs (85%; *n* = 2), the CS-POR loaded NPs showed lesser degradation (65%; *n* = 3) and improve release profile till 12 hours when studied at 2, 4, 6, 8, 10, and 12 h intervals (Figure 10(b)). In addition there were no significant differences found between the degradation of CS-POR PEC (65%; *n* = 3) and CS-POR-TPP PEC (69%; *n* = 1) which indicates the protective efficiency of optimized formulation in gastric environment even after 12 hrs (Figure 10(b)). This might be due to the accumulation/entrapment of AmB in compact matrix shell/space which cannot be hydrolyzed easily. These findings suggested that CS-POR PEC NPs can be utilized as sustained release gastroretentive delivery system for fungicidal drug like AmB.

### 3.9. *In Vitro* Fungal Activity

The prepared AmB nanoformulations exerted significant (*P* ≤ 0.001) higher antifungal activity in comparison with the reference drug and marketed formulations (Table 2). The IC_50_ values of pure AmB (oral grade), Fungizone, and Ambisome against* A. fumigatus*,* A. niger*,* A. flavus*, and* C. albicans* were evaluated and compared with prepared formulations as mentioned in Table 2. CS-POR blank NPs have shown no antifungal activity as they were inactive on the panel of test organisms (IC_50_, >182 *μ*g/mL). Among all the preparations AmB loaded CS-POR formulations have shown better activity. Moreover the antifungal activity of CS NPs was lower than CS-POR-AmB NPs but was found to be significant in inhibiting the growth of all four fungal strains. These preparations even maintain their inhibition potential effectively till 48 hours as mentioned in Table 2. Similarly TPP based formulations exerted almost similar antifungal activity as exerted by CS-POR-AmB.

Thus CS-POR-AmB formulation with the lowest IC_50_ was the most effective formulation against the four fungal species (Table 2). CS-POR-AmB was 23-fold more active than Ambisome when tested in* A. fumigatus* and around 12- to 15-fold more active than* A. niger*,* A. flavus*, and* C. albicans.* The developed nanoformulations were more effective (*P* ≤ 0.001) against three* Aspergillus* spp. than Ambisome and Fungizone. It is also important to point out that many drug delivery systems are more active* in vivo* than* in vitro*. This is the case of Ambisome and it has been reported in many studies [43].

### 3.10. *In Vitro* Hemolytic Study

**Figure 11:**
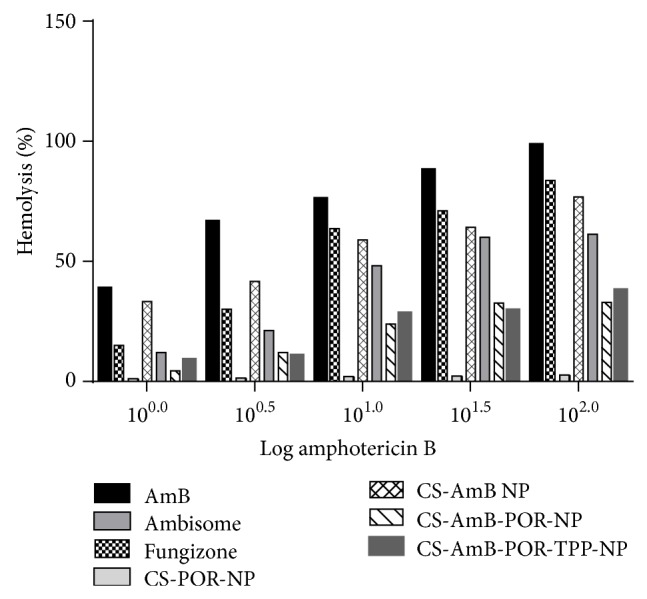
*In vitro* cytotoxicity test (hemolysis test) for AmB, Fungizone, Ambisome, CS blank, CS-AmB, and various CS-POR based NPs. Values represent mean ± SD (*n* = 3).

Hemolytic toxicity data was evaluated by comparing the hemolytic toxicity of standard drug (AmB) with marketed and prepared nanoformulations. It has been observed that hemolytic toxicity was found to be maximum in the samples of standard drug (AmB) and minimal in the blank CS-POR. The hemolytic toxicity in decreasing order is mentioned as follows: AmB > Fungizone > CS-AmB > CS-POR-AmB > Ambisome > CS-POR. Blank NPs exerted no hemolysis in the tested concentration range. Hemolytic activity was also absent for Ambisome, even at the highest AmB concentration. PEC was nontoxic up to concentration of 55.5 *μ*g/mL and shows 10 times reduction in hemolysis of formulations in comparison to plain AmB. The percentage of hemolysis observed in CS-POR-AmB formulations was 4.1% ± 1.1% in comparison with standard 39.86% ± 0.12% (Figure 11).

### 3.11. *In Vivo* Toxicity Studies

#### 3.11.1. *In Vivo* Hemolytic Study

Hematological parameters (RBC and WBC, hemoglobin, hematocrit, and platelet count) were evaluated at the doses 1, 4, and 8 mg/kg (AmB equivalent) for standard drug solution (AmB) solution, Ambisome, CS-AmB, CS-POR-AmB, and CS-POR-AmB-TPP (Table 3). It has been observed that at similar dose administration of CS-POR NPs (blank) suddenly increase the platelet count (433 ± 33) which was three times greater than plain AmB solution. Even value of platelet count for CS-POR-AmB (389 ± 41) was larger than control (372 ± 21). Except platelet count there was no significant difference seen between CS-POR blank NPs and control. It has been also found that throughout the hematological studies there was no significant difference between the values of CS-POR-AmB and CS-POR-AmB-TPP. Similarly no significant difference was found between marketed preparations Fungizone and plain AmB values whereas Ambisome values were comparable with CS-AmB NPs. Otherwise there were significant differences (*P* > 0.05) found in all blood parameters for plain AmB solution (Table 3) when compared with control.

#### 3.11.2. *In Vivo* Determination of Blood Urea Nitrogen and Serum Creatinine

**Figure 12:**
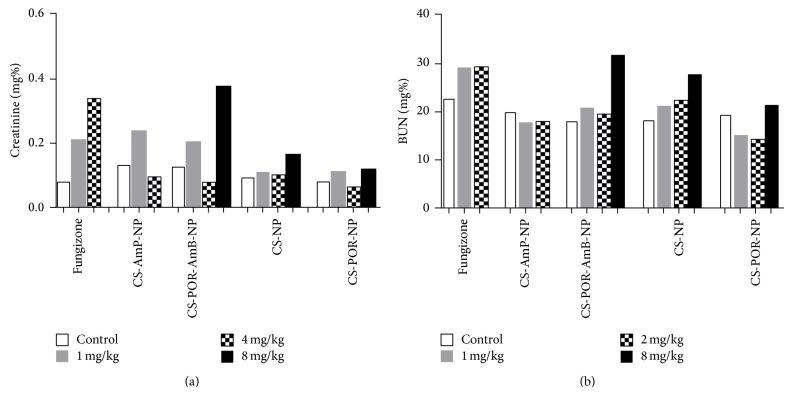
Renal toxicity of Fungizone, CS-AmB-NP, CS-POR-AmB-NP, CS-NP (blank), and CS-POR-NP (blank) nanoparticles. The concentrations of (a) BUN and (b) Creatinine in serum were determined at 1 mg/kg, 4 mg/kg, and 8 mg/kg dose intervals. From 8 mg/kg of Fungizone and CS-AmB NPs no survival of mice was observed. Values represent mean ± SD (*n* = 3).

Renal toxicity was examined by treating mice with normal to high dose of Fungizone, CS-AmB, CS-POR-AmB, CS, and CS-POR NPs formulations at 1, 4, and 8 mg/kg (equivalent to amphotericin B). Serum was collected from the survived mice on the 14th day and analyzed for blood urea nitrogen (BUN) and creatinine levels (Figures 12(a) and 12(b)). It has been reported that among all preparations POR based nanoformulations (up to its 4 mg/kg dose) have not shown a significant difference between creatinine and BUN level against the control. At highest dose (8 mg/kg) of CS-POR-AmB nanoparticles mice were survived, whereas Fungizone administration resulted in significant difference in creatinine and BUN level against the control. Thus in contrast with Fungizone^®^ (shown maximum renal toxicity at 1 mg/kg), CS-POR blank NPs have shown no renal toxicity at 8 mg/kg. The renal toxicity at maximum dose is represented as follows: Fungizone > CS-AmB > CS-POR-AmB > CS > POR.

## 4. Discussion

Porphyran (isolated from various species of* Porphyra*) is a new emerging sulfated polysaccharide which is recently introduced in nanoparticulate delivery system for its excellent biological and pharmaceutical properties [20, 21, 42]. Due to its nontoxic, biodegradable, biocompatible, frequent gelling, and excellent emulsifying properties it can act as a nanocarrier system for antifungal drugs [22, 23]. Best advantages of this polymer are the presence of sulfur group which not only enhances its antifungal activity [44–46] but also imparts a negative charge on the polymer, making it much more suitable candidate for nano-PEC. Apart from its various advantages there are some serious drawbacks of this polymer such as its high molecular weight and uneven mass (charge) distribution [27]. To reduce the molecular weight and convert in to uniform mass it can be hydrolyzed by means of either alkali or acidic or enzymatic or radical hydrolysis [31, 42]. But hydrolysis procedure should be carefully selected to avoid the desulfation and unusual transformation of functional groups. One of the best known hydrolysis methods is NaBr reduction method [31]. Several reports suggested that NaBr causes desulfation [21, 45]. Therefore, in present study NPOR (isolated from red alga,* Porphyra vietnamensis,* yielding 28.9% of NPOR which contains 16.36% sulfate content and 21.1% of 3,6-anhydrogalactose) was reduced at 10°C by using lowest concentration of NaBr to avoid its desulfation during this procedure (modified Ishihara et al. (2005) method) [31]. Alkali reduction was confirmed by FTIR which does not cause desulfation and any major group transformation (Figure 7). Concentration of sulfur and AGR was again determined after hydrolysis which suggested 16% reduction in the quantity of sulfur and 33% reduction in AGR. After reduction in molecular weight it has been observed that the emulsifying, gelling, and viscometric properties were improved (data not shown) [23]. Therefore here in this work we explored a novel PEC technique by using CS as a cationic (positive) polysaccharide and POR as an anionic polysaccharide (Figure 1), with an objective to form stable oral nanoformulation of AmB. The NPs were prepared and optimized to minimize particle size and maximize percent drug entrapment efficiency using three-factor three-level (3^3^) central composite designs. Sulfated polysaccharide assay confirmed the presence of POR : CS concentration in 7 : 4 (%w/w) ratios which signifies the higher concentration of POR in CS-POR NPs (Figure 4). During preparation it has been observed that these two polyions strongly interact with each other to form a clear turbid solution. FTIR analysis of CS-POR demonstrates the spectral shifts in the sulfate and amine regions which confirm the existence of a strong electrostatic interaction between the POR sulfate groups and CS amine groups (Figure 7). One positive aspect of this method is that surfactants and stabilizers were not utilized anywhere in the formulation.* In vitro* dissolution and degradation (Figures 9 and 10) studies results (at pH 1.2 and 7.4) suggested the stability of this formulation at wide pH range which was further confirmed by the UV spectrophotometric method (incubation of the present formulation at 21°C till one week, does not show UV change (data not shown)). The probable reasons were based on sulfate and AGR mediated drug release as explained below.

Hemolytic toxicity data demonstrated that AmB loaded CS-POR (nontoxic up to 55.5 *μ*g/mL) has shown minimal hemolytic toxicity when compared against standard drug, AmB, Fungizone, and CS-AmB (Figure 11). On the other side hematological parameters evaluated results (Table 3) suggested that administration of POR based nanoformulations suddenly increases the platelet (Platelet count: blank (433 ± 33) > control (372 ± 21) > CS-POR-AmB (389 ± 41)) and very slight decrease in RBC, WBC, Hb, and hematocrit values was observed against control. These findings could be explained on the basis of the inhibition of complement activation, extracellular matrix-degrading enzymes, and tumor cell adhesion to P-selectin by SPs which may lead to platelet stimulation and reduced the extent of hemolysis [47]. Platelet activation could be either due to the high degree of sulfation or the presence of high amount AGR per moles of the samples.

**Figure 13:**
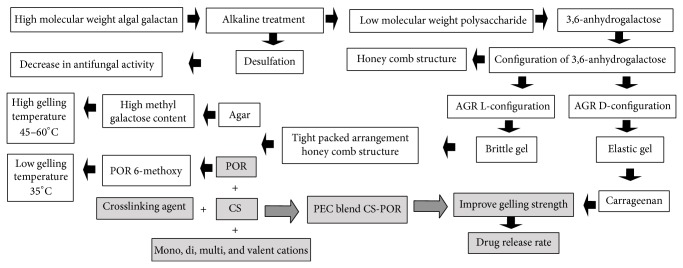
(1) Justification on probable relationship between structural features of POR (brittle gelling properties) and better drug release rate profile (improvement strategies such as CS blend) caused by improving the gel strength (by CS-POR blend), to improve the effectiveness of whole formulation against fungal strains [15–17]. (2) Role of alkaline treatment in conversion of high to low mol. wt. sulphated polysaccharide (with conversion of L-galactose 6-sulfate residue to 3,6 anhydro form) which improves the gelling strength followed by the release rate profile of matrix [15–17].

According to the earlier reports AmB shows increased solubility but relatively low stability at acidic pH. Entrapment of AmB in PEC could be proved to be a good strategy, as polymerization and high charge density of the two oppositely charged polyions, their associated pH, and ionic strength play an important role in disaggregation of the aggregates to improve the solubility of the drug. Various reports suggested the existence of AmB in monomeric or aggregated state which is dependent on solvent polarity and AmB concentration (Figure 5). Beyond a critical concentration, the AmB exists in either aggregated or micellar form and thus shows different types of activity. The factors which cause disturbance in equilibrated forms of AmB in solution state are responsible for change in the overall activity of the drug. Furthermore formation of aggregates (self-aggregation or association with other compounds) leads to change in molecular conformation/spatial arrangement which form the underlying cause behind the renal toxicity of AmB (Figure 13). Using spacing solvents, stabilizers, and surfactant could be a good strategy to eliminate these problems; however, these techniques sometimes cause toxicity and incompatibility problems during the formulation (Figures 2 and 13). The role of present formulation in preventing these problems can be explained on the basis of high molecular weight associated high sulfate: AGR in comparison with CS amino group which imparts high negative charge associated high positive zeta potential. The existence of high electric charge on the surface of the AmB loaded CS-POR could create strong repulsive forces among particles to prevent aggregation of the CS-POR and favors the high stability of CS-POR formulation. This aggregation/molecular structure of AmB in the present formulations was confirmed by UV-Vis spectroscopy (300–450 nm). Thus it has been clarified that two molecular conformations (aggregated/monomeric) of AmB produce two distinct absorption spectra [25]. First is AmB monomeric state which gives a spectrum constituting of four well-separated bands at 344, 365, 385, and 410 nm and second is AmB aggregated state which gives broad single band at 328 accompanied by decreasing in intensity at 365, 385, and 410 nm. According to our current study it has been proven that the UV absorption spectrum (Figure 5) of our prepared formulation was similar to Fungizone which clearly indicates the presence of AmB in aggregated state [25]. The aggregated state of AmB was further confirmed by peak ratios (first peak (I) to the fourth peak (I/IV) ratio < 0.25 represents monomeric AmB and ratio ≥ 2.0 corresponds and characterizes the highly aggregated species) which were represented as follows: Ambisome (4.2) > CS-POR-AmB (3.5) > Fungizone (3.3). Due to the existence of either repulsive forces or small size and uniform shape of NPs, this type of aggregation is relatively weak. Explanation related with efficient* in vitro* fungal activity was purely based on either presence of high concentration of sulfur in antimicrobial SPs (confirmed by SP assay and further sulfur test) which could synergy the antifungal activity or better release rate (confirmed by dissolution studies) of AmB in prepared NPs (Figures 4 and 9). However the clear reason behind this is still unknown. Molecular studies of transfer mechanism of AmB from PEC carriers to fungal strain can shed light on this.

Renaltoxicity and nephrotoxicity were examined on the basis of creatinine and urea nitrogen level in blood after the administration of various formulations. The aggregated form of AmB NPs in AGR matrix of POR and CS has shown relatively lesser renal toxicity. The renal toxicity at maximum dose is represented as follows: Fungizone > CS-AmB > CS-POR-AmB > CS > POR (Figure 12). This could be again explained on the basis of release rate of AmB controlled by AGR and sulfur present in POR. AGR induces gelling property in algal galactans (Figure 13). At elevated temperature the gelling strength is dependent on configuration of AGR which is further dependent on the formation of honey comb structure (precursor stage in gelling of algal galactans) (Figure 13). For example, carrageenan type galactans (containing AGR of D-configuration) gel by forming the tight honeycomb structure and hence provide more elasticity but POR comes under agar type galactans (containing AGR of L-configuration), built up of more tight and homogeneous network resulting in brittle and strong gel [15–17]. Furthermore, even after the presence of 6-O-methyl and sulfur groups (that may lead to elastic-brittle gel textures), POR in comparison with agar (contains high methyl galactose and gels at 45–55°C) gels at 30–35°C [15–17]. Elimination of sulfur groups by alkaline treatment increased the AGR dependent gelling strength but declined its antifungal activity (Figure 13). These are the considerable factors which determine the drug release rate from the matrix. CS- (elasticity-) POR (stiffness) complexation improves mechanical and gelling strength, release rate, and tolerance of whole formulation against wide pH range (Figure 13). Thus current preparation was more suitable for gastroretentive release of AmB.

## 5. Conclusion

AmB is among the oldest polyene derivatives that is still therapy of choice for systemic mycosis in various immunocompromised patients. Fungizone (AmB deoxycholate) is the most popular marketed parenteral formulation which is also associated with significant toxicities. Thus due to high toxicities and poor prognosis there is a great need of safe and effective AmB formulation. In current studies CS-POR PEC was prepared and optimized (central composite design) using AmB as a model drug. The designed complex significantly improved this activity while addition of TPP was not able to produce significant changes. The improved activity may be attributed to the presence of sulfur and AGR in the backbone of POR which when blends with CS provides better release and lesser degradation, reduces toxicity, and may finally lead to enhanced antifungal activity.

## Supplementary Material


*Porphyra vietnamensis* contains therapeutic polysaccharide called as Porphyran. There are various factors that influence the molecular weight of this red alga polysaccharide. Alkaline hydrolysis yields Porphyran with much better physical and chemical properties. Negative charge of this polysaccharide can be utilized for the preparation of polyelectrolyte complex. Therefore in this process PEC was prepared by using POR as negative and chitosan as positive polymer. Stable AmB nanoparticles were developed by polyelectrolyte complexation technique in the presence of TPP. These nanoparticles causes lysis of fungi as illustrated in supplementary data.

## Figures and Tables

**Table 1:** Composition, particle size (P), zeta potential (*Z*), and maximum percentage of drug entrapped (PDE) of various batches of Chitosan-POR nanoparticle formulations as per experimental design.

F. code	Trial no.	Independent variables (coded factor levels)			Dependent variables		
		*X*1: CS conc. (%w/v)	*X*2: POR conc. (%w/v)	*X*3: TPP conc. (%w/v)	P (nm)	*Z* (mV)	PDE (%)
22	1	0.04	0.025	0.02	165	36.98	62.23
24	2	0.04	0.025	0.02	174	34.57	67.15
9	3	0.006364	0.025	0.02	111	24.44	68.11
12	4	0.04	0.033409	0.02	212	33.23	65.22
3	5	0.02	0.03	0.01	170	29.22	70.11
10	6	0.073636	0.025	0.02	360	58.33	68.11
20	7	0.04	0.025	0.02	179	35.86	77.22
7	8	0.02	0.03	0.03	125	26.62	82.29
17	9	0.04	0.025	0.02	179	37.12	69.23
21	10	0.04	0.025	0.02	172	36.33	71.18
18	11	0.04	0.025	0.02	186	34.89	56.67
15	12	0.04	0.025	0.02	168	39.22	74.76
23	13	0.04	0.025	0.02	174	35.11	63.99
8	14	0.06	0.03	0.03	240	38.46	81.11
5	15	0.02	0.02	0.03	112	21.88	87.22
2	16	0.06	0.02	0.01	310	48.74	61.58
19	17	0.04	0.025	0.02	185	32.67	68.29
13	18	0.04	0.025	0.003182	255	43.11	66.22
16	19	0.04	0.025	0.02	181	28.44	73.11
4	20	0.06	0.03	0.01	340	51.11	72.89
6	21	0.06	0.02	0.03	261	45.11	76.62
14	22	0.04	0.025	0.036818	158	25.12	82.23
1	23	0.02	0.02	0.01	133	23.11	76.33
11	24	0.04	0.016591	0.02	198	27.33	60.39

**Table 2:** *In vitro* antifungal activity of AmB nanoformulations against commercial products and plain AmB (*n* = 3 minimum). IC_50s_ of nanoformulations are expressed as equivalent concentration of AmB.

AmB nanoformulation and model drug	*In vitro *activity, IC_50_ ± SD (*μ*g/mL) on							
	*A. fumigatus *		*A. niger *		*A. flavus *		*C. albicans *	
	24 h	48 h	24 h	48 h	24 h	48 h	24 h	48 h
AmB	1.44 ± 0.11	1.38 ± 0.01	1.68 ± 0.01	1.63 ± 0.07	1.33 ± 0.11	1.27 ± 0.33	0.59 ± 0.03	0.55 ± 0.08
Fungizone	0.49 ± 0.31	0.41 ± 0.02	0.57 ± 0.03	0.53 ± 0.01	0.41 ± 0.02^∗∗∗^	0.39 ± 0.14	0.29 ± 0.021	0.26 ± 0.013
AmBisome	1.92 ± 1.03	1.84 ± 0.02^∗∗∗^	2.01 ± 1.23	1.93 ± 0.02	1.69 ± 0.33^∗∗∗^	1.56 ± 0.02^∗∗∗^	0.52 ± 0.41	0.50 ± 1.14
CS-AMB-NP	0.50 ± 0.01	0.45 ± 0.03	0.74 ± 011	0.68 ± 0.03	1.56 ± 0.02	1.51 ± 1.11	0.26 ± 1.23	0.21 ± 0.01
CS-AMB-POR	0.10 ± 0.01	<0.08^∗∗∗^	0.17 ± 0.01	0.16 ± 0.02	0.12 ± 0.02	0.10 ± 0.03^∗^	0.08 ± 0.03^∗∗∗^	<0.04^∗∗^
CS-AMB-POR-TPP	0.16 ± 0.02	0.15 ± 1.11	0.11 ± 0.01	0.10 ± 1.37	0.11 ± 0.02	0.09 ± 0.01	0.08 ± 0.02^∗∗∗^	<0.03^∗∗∗^

*n* = 6 in each group. Values are expressed as mean ± S.E.M. Data was analyzed by one-way ANOVA followed by Tukey's post hoc test.

^∗^Significant difference with AmB, pb0.05.

^∗∗^Significant difference with AmB, pb0.01.

^∗∗∗^Significant difference with AmB, *P* ≤ 0.001.

**Table 3:** Dose (1, 4, and 8 mg/kg/day of AmB) related *in vivo* hematological responses of prepared formulations against marketed and reference drug.

Parameters	Doses (mg/kg/day)	Std. drug sol.	Fungizone	Ambisome	CS-AMB	CS-POR-AMB	CS-POR-AMB-TPP	Control	Blank
RBCs (×10^6^/*μ*L)	1	5.21 ± 0.3^∗^	5.77 ± 0.2	6.16 ± 1.3	6.18 ± 1.8	7.12 ± 1.5	7.08 ± 0.27	7.96 ± 1.8	8.12 ± 1.2
	4	4.11 ± 1.2	4.81 ± 1.3	5.21 ± 1.1	5.53 ± 1.7	6.78 ± 1.8	6.31 ± 1.6		
	8	3.71 ± 1.1^∗^	4.07 ± 1.3^∗^	4.14 ± 0.3	4.11 ± 0.41	5.72 ± 1.2	5.22 ± 1.0		

WBCs (×10^3^/*μ*L)	1	4.7 ± 0.3^∗^	5.1 ± 1.6	6.0 ± 1.1	5.9 ± 1.4	6.2 ± 0.7	5.8 ± 1.6	6.2 ± 1.1	5.9 ± 0.2
	4	4.4 ± 1.3	4.6 ± 1.3	5.5 ± 1.4	5.7 ± 0.6	5.7 ± 1.2	5.3 ± 0.1		
	8	3.8 ± 0.8^∗^	4.8 ± 1.7^∗^	5.1 ± 1.8	4.9 ± 0.3	5.4 ± 1.1	5.2 ± 0.2		

PL (×10^3^/*μ*L)	1	134 ± 23	178 ± 11	275 ± 18	281 ± 26	389 ± 41^∗^	371 ± 33	372 ± 21	433 ± 33^∗^
	4	106 ± 28	144 ± 27	239 ± 23	237 ± 1.3	274 ± 11^∗^	266 ± 23		
	8	92 ± 11^∗^	116 ± 13^∗^	191 ± 16	182 ± 12	211 ± 31^∗^	207 ± 17		

HT (%)	1	30.2 ± 2.4	32.1 ± 2.2	35.2 ± 3.1	34.1 ± 2.8	38.7 ± 3.4	38.2 ± 2.4	42.1 ± 3.1	41.1 ± 2.7
	4	28.1 ± 3.1	30.7 ± 1.1	33.5 ± 2.1	31.1 ± 1.9	36.6 ± 3.1	35.2 ± 3.3		
	9	26.1 ± 2.5^∗^	29.1 ± 2.6^∗^	30.2 ± 1.7	29.1 ± 2.3	35.1 ± 1.7	34.1 ± 1.8		

Hb (g/dL)	1	12.1 ± 1.3	13.2 ± 0.3	13.8 ± 0.2	13.3 ± 0.5	14.6 ± 3.2^∗^	14.3 ± 1.2	15.2 ± 1.4	15.1 ± 1.2
	6	11.2 ± 1.1	12.4 ± 1.4	12.6 ± 1.6	12.1 ± 1.8	13.7 ± 0.3	13.6 ± 1.6		
	9	10.3 ± 0.2^∗^	11.1 ± 0.2	11.8 ± 0.1	11.7 ± 1.2	12.8 ± 1.3	12.7 ± 0.1		

RBCs (red blood cells); WBCs (white blood cells); Hb (hemoglobin); PL (platelet); HT (hematocrit).

^∗^Significant (*P* < 0.05, all groups compared with control). *n* = 6 in each group. Values are expressed as mean ± S.E.M. Data was analyzed by one-way ANOVA followed by Tukey's post hoc test.

## Abbreviations

AGR: 3, 6-Anhydrogalactose residue
AmB: Amphotericin B
CS: Chitosan
CS-POR: Chitosan-porphyran complex
DMSO: Dimethylsulfoxide
DSC: Differential scanning calorimetry
FTIR: Fourier transform infrared spectroscopy
Hb: Hemoglobin
NaBr: Sodium borohydride
NPOR: Natural porphyran
NPs: Nanoparticles
PBS: Phosphate-buffered saline
PEC: Polyelectrolyte complex
POR: Sodium borohydride treated porphyran
POR: Porphyran
RBCs: Red blood cells
SGF: Simulated gastric fluid
SPs: Sulfated polysaccharides
TEM: Transmission electron microscopy
TPP: Tripolyphosphate pentasodium
WBCs: White blood cells.
