# Supplementary material for: Significance of Algal Polymer in Designing Amphotericin B Nanoparticles
Source: ScientificWorldJournal. 2014 Nov 12;2014:564573. doi: 10.1155/2014/564573 (PMC4244925; doi:10.1155/2014/564573)
Supplement: Supplementary file 1 — Porphyra vietnamensis contains therapeutic polysaccharide called as Porphyran. There are various factors that influence the molecular weight of this red alga polysaccharide. Alkaline hydrolysis yields Porphyran with much better physical and chemical properties. Negative charge of this polysaccharide can be utilized for the preparation of polyelectrolyte complex. Therefore in this process PEC was prepared by using POR as negative and chitosan as positive polymer. Stable AmB nanoparticles were developed by polyelectrolyte complexation technique in the presence of TPP. These nanoparticles causes lysis of fungi as illustrated in supplementary data. [file 564573.f1.pdf]

GRAPHICAL ABSTRACT

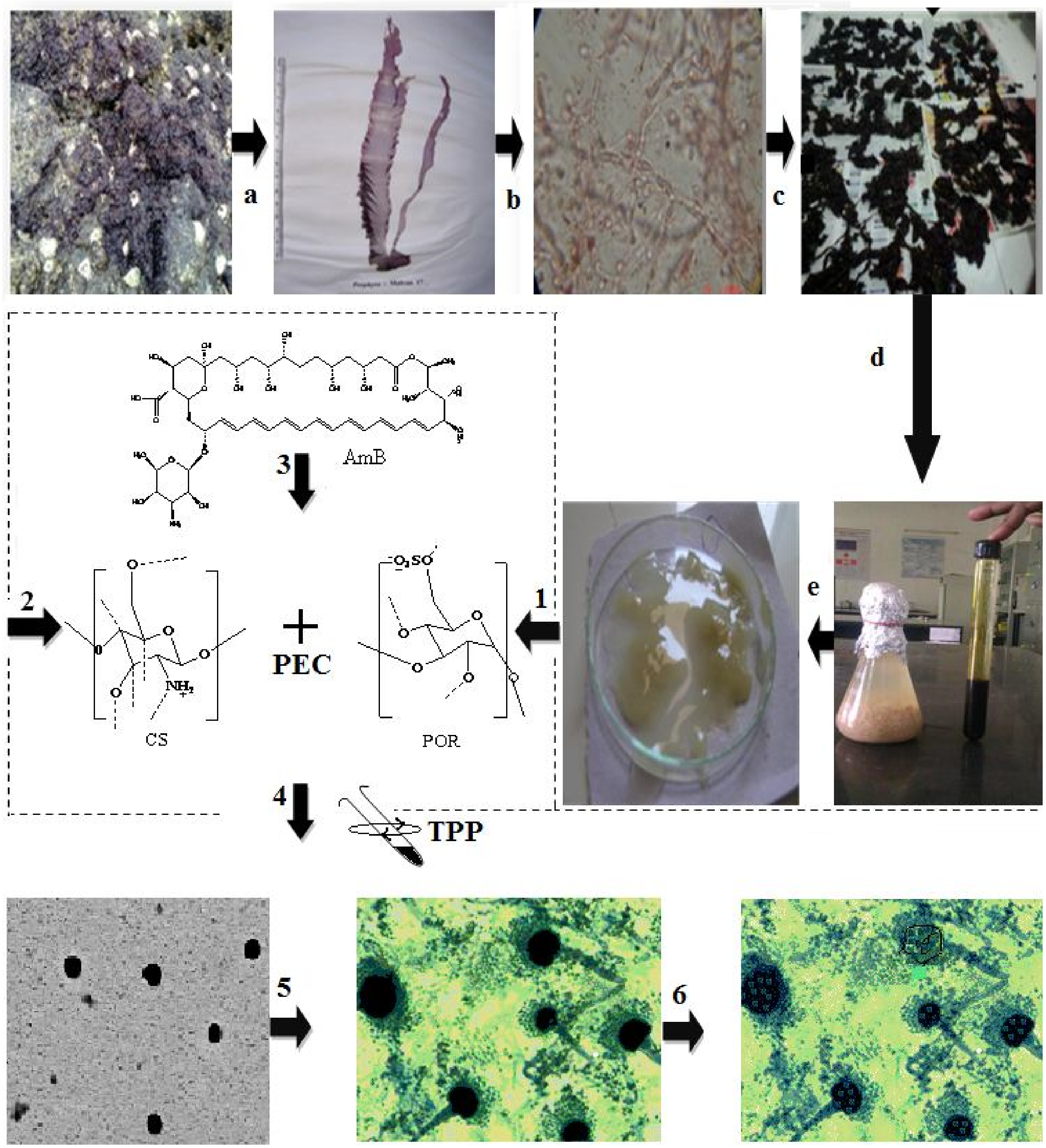

Porphyra from rock (a) was collected and identified macroscopically (b) and microscopically (c). After hot air drying *Porphyra* was alkali hydrolyzed (d) and Porphyran, POR (porphyran) was isolated. By using POR (e) as negative (1) and chitosan (CS) as positive polymer (2) stable AmB (3) nanoparticles were developed by PEC (polyelectrolyte complexation technique) in the presence of TPP(4). These NPs causes lysis of fungi (5&6) as illustrated above.
